# A Case of Fulminant Immune‐Related Enterocolitis During Immune Checkpoint Inhibitor Therapy for Metastatic Renal Cell Carcinoma

**DOI:** 10.1002/iju5.70181

**Published:** 2026-04-16

**Authors:** Kasumi Kanai, Takayuki Sumiyoshi, Hiroki Kitamoto, Yuki Teramoto, Yuta Mine, Jin Kono, Kimihiko Masui, Takayuki Goto, Ryoichi Saito, Takashi Kobayashi

**Affiliations:** ^1^ Department of Urology Kyoto University Hospital Kyoto Japan; ^2^ Department of Gastroenterology Kyoto University Hospital Kyoto Japan; ^3^ Department of Diagnostic Pathology Kyoto University Hospital Kyoto Japan

**Keywords:** diarrhea, immune checkpoint inhibitors, immune‐related enterocolitis, metastatic renal cell carcinoma, nivolumab

## Abstract

**Introduction:**

Combination therapy with immune checkpoint inhibitors (ICIs) has become a standard treatment for metastatic renal cell carcinoma (mRCC). However, ICIs may also cause immune‐related adverse events. We report a case of mRCC that developed fulminant immune‐related enterocolitis.

**Case Presentation:**

A 79‐year‐old man received ICIs therapy for mRCC and was urgently hospitalized because of worsening diarrhea. Despite the initiation of steroid therapy, circumferential intestinal necrosis developed, necessitating subtotal colectomy. The patient subsequently developed cytomegalovirus‐ and Epstein–Barr virus‐associated enterocolitis and died from extensive intestinal necrosis caused by severe thrombosis secondary to disseminated intravascular coagulation.

**Conclusion:**

Persistent diarrhea during ICIs therapy requires prompt evaluation and specialist consultation, even when symptoms appear mild, as they may signal immune‐related enterocolitis. Clinicians should also remain vigilant for infectious complications, which can exacerbate the severity of immune‐related enterocolitis and lead to life‐threatening outcomes.

## Keynote Message

We report a case of immune‐related enterocolitis that progressed to colonic necrosis and small intestinal perforation during immune checkpoint inhibitor therapy for metastatic renal cell carcinoma. High‐dose steroid therapy for immune‐related enterocolitis may have reactivated cytomegalovirus and Epstein–Barr virus, further worsening the disease course. When diarrhea occurs during immune checkpoint inhibitor therapy, clinicians should initiate prompt management, including early consultation with a gastroenterologist, even while symptoms remain mild.

**Abbreviations and Acronyms**
CMV: cytomegalovirus
CT: computed tomography
CTCAE: Common terminology criteria for adverse events classification
DIC: disseminated intravascular coagulation
EBV: Epstein–Barr virus
ICIs: immune checkpoint inhibitors
irAEs: immune‐related adverse events
mRCC: metastatic renal cell carcinoma
PCR: polymerase chain reaction

## 1 Introduction

Several clinical trials have shown that combination therapies involving immune checkpoint inhibitors (ICIs) improve progression‐free and overall survival in patients with metastatic renal cell carcinoma (mRCC) [1]. However, ICIs can lead to specific complications known as immune‐related adverse events (irAEs), which differ from those caused by cytotoxic chemotherapy [2, 3]. irAEs may affect any organ system, with gastrointestinal toxicity being second only to dermatological toxicity in frequency [4]. We report the case of a patient with mRCC who developed fulminant immune‐related enterocolitis after 4 years of nivolumab therapy.

## 2 Case Presentation

A man in his 70s with mRCC, lung metastases, and peritoneal dissemination was treated with nivolumab as second‐line therapy. Nivolumab treatment maintained stable disease at the metastatic site for a long period. After 4 years of treatment, he developed diarrhea occurring two to three times daily. Lower gastrointestinal endoscopy performed at another hospital revealed rectal inflammation, but conservative management with intestinal regulators was continued. Six months later, his diarrhea worsened to more than 10 episodes per day, accompanied by anorexia, leading to emergency hospitalization.

**FIGURE 1:**
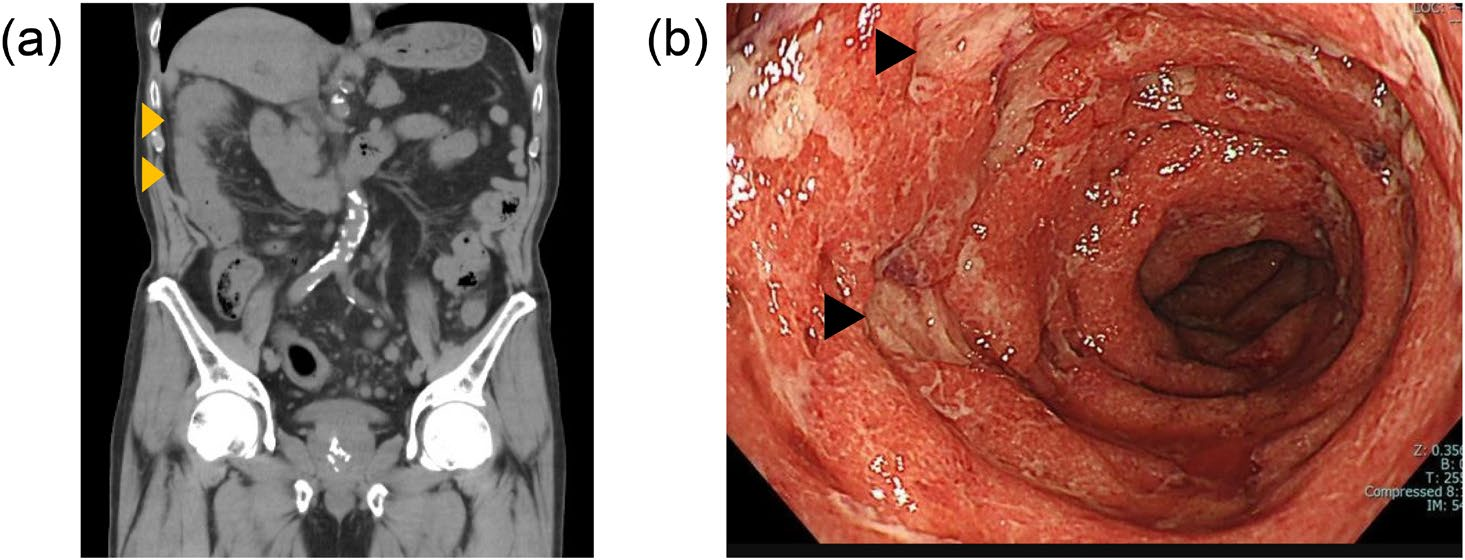
Imaging findings at admission. (a) Abdominal plain computed tomography showing bowel wall thickening throughout the colon (orange arrows). (b) Lower gastrointestinal endoscopy showing Grade 3 ulcerative lesions throughout the colon (black arrows).

Blood tests at admission showed an elevated white blood cell count of 14 770/μL and a C‐reactive protein level of 9.98 mg/dL. Abdominal computed tomography (CT) demonstrated diffuse bowel wall thickening throughout the colon (Figure 1a). Lower gastrointestinal endoscopy revealed ulcerative lesions extending across the colon (Figure 1b), and tissue biopsy showed crypt abscesses and apoptotic epithelial cells. Although cytomegalovirus (CMV) polymerase chain reaction (PCR) testing of the tissue was weakly positive, serum CMV antigens were not detected.

**FIGURE 2:**
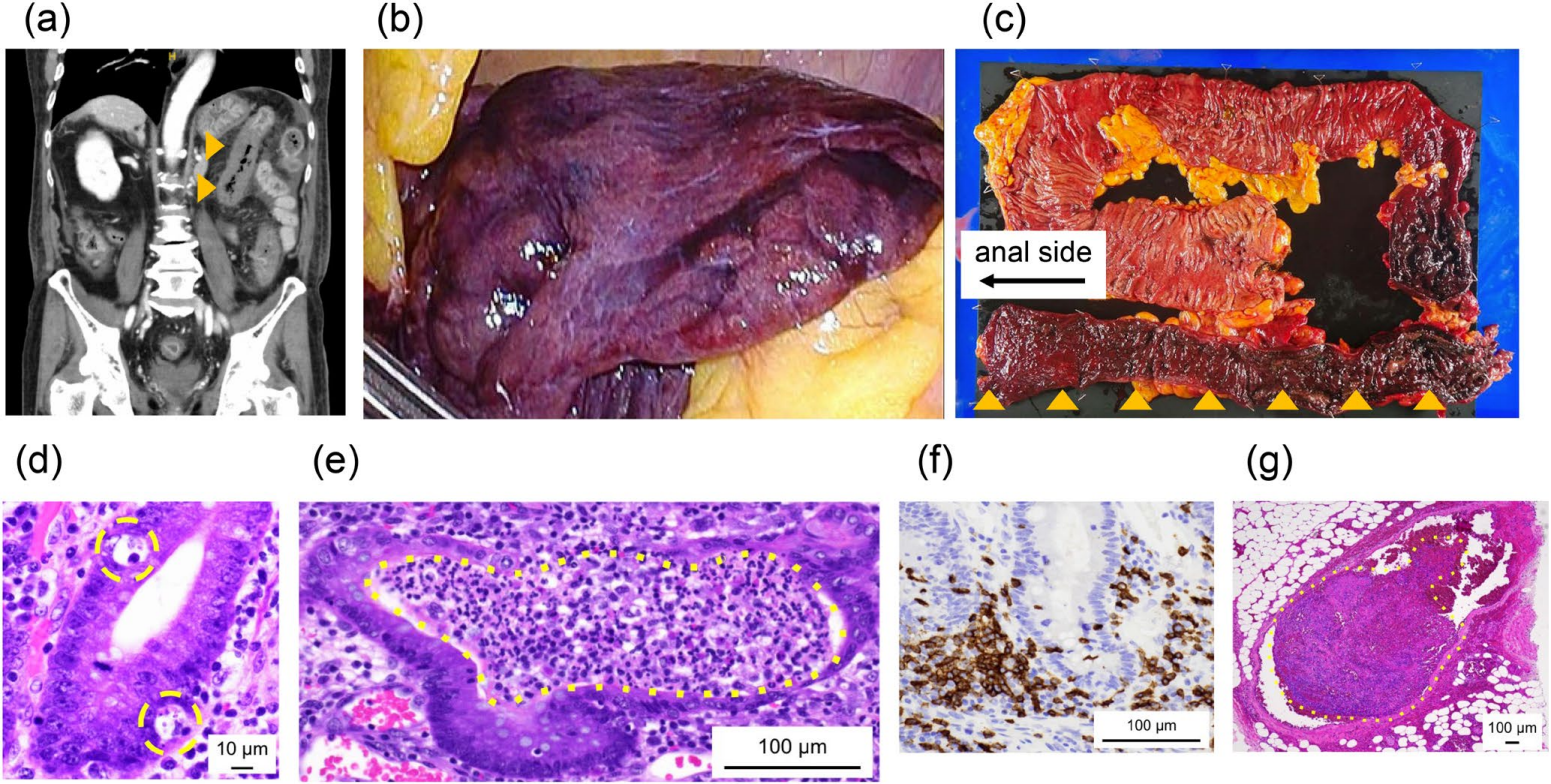
Radiologic, surgical, and histopathological findings of colonic necrosis. (a) Contrast‐enhanced abdominal computed tomography showing wall thickening and poor contrast enhancement from the descending colon to the sigmoid colon, suggestive of necrosis (orange arrows). (b) Intraoperative findings showing black discoloration of the colonic wall extending from the splenic flexure to the rectum. (c) Macroscopic view of the resected specimen lumen showing black discoloration from the splenic flexure to the rectum (orange arrows). (d) Scattered apoptotic epithelial cells are seen in the mucosa (yellow circles). (e) The crypts are irregularly dilated with abscess formation (yellow circles). (f) The lamina propria shows dense lymphoplasmacytic infiltration, with CD8 immunostaining highlighting numerous cytotoxic T cells. (g) The submucosa contains venous thrombi (yellow circles), indicating secondary ischemic change.

Based on the clinical course and the results of the two imaging examinations, the patient was diagnosed with immune‐related colitis. In consultation with the irAEs care expert team, steroid therapy (prednisolone 2 mg/kg) was initiated on the third day of hospitalization. This treatment promptly relieved the diarrhea; however, on the tenth day of hospitalization, the symptoms worsened, and blood testing demonstrated an elevated inflammatory response. At that time, serum CMV antigen became detectable, leading to a diagnosis of concurrent CMV colitis, and ganciclovir therapy was initiated. On the 15th day of hospitalization, the patient developed acute severe abdominal pain, and contrast‐enhanced CT revealed bowel wall thickening and reduced contrast enhancement from the descending colon to the sigmoid colon, findings consistent with intestinal necrosis (Figure 2a). An emergency subtotal colorectal resection was therefore performed. Intraoperative findings showed black discoloration of the colon wall extending from the splenic flexure to the rectum, consistent with necrosis, and the lumen of the resected specimen was macroscopically blackened (Figure 2b,c). Histopathological examination revealed crypt abscesses and apoptotic epithelial cells, along with infiltration of lymphocytes and plasma cells in the mucosal lamina propria. Immunohistochemical staining demonstrated CD8‐positive cells, supporting a pathological diagnosis of immune‐related colitis (Figure 2d–f). In addition, numerous venous thrombi were identified in the intestinal submucosa, indicating ischemic changes within the bowel (Figure 2g).

**FIGURE 3:**
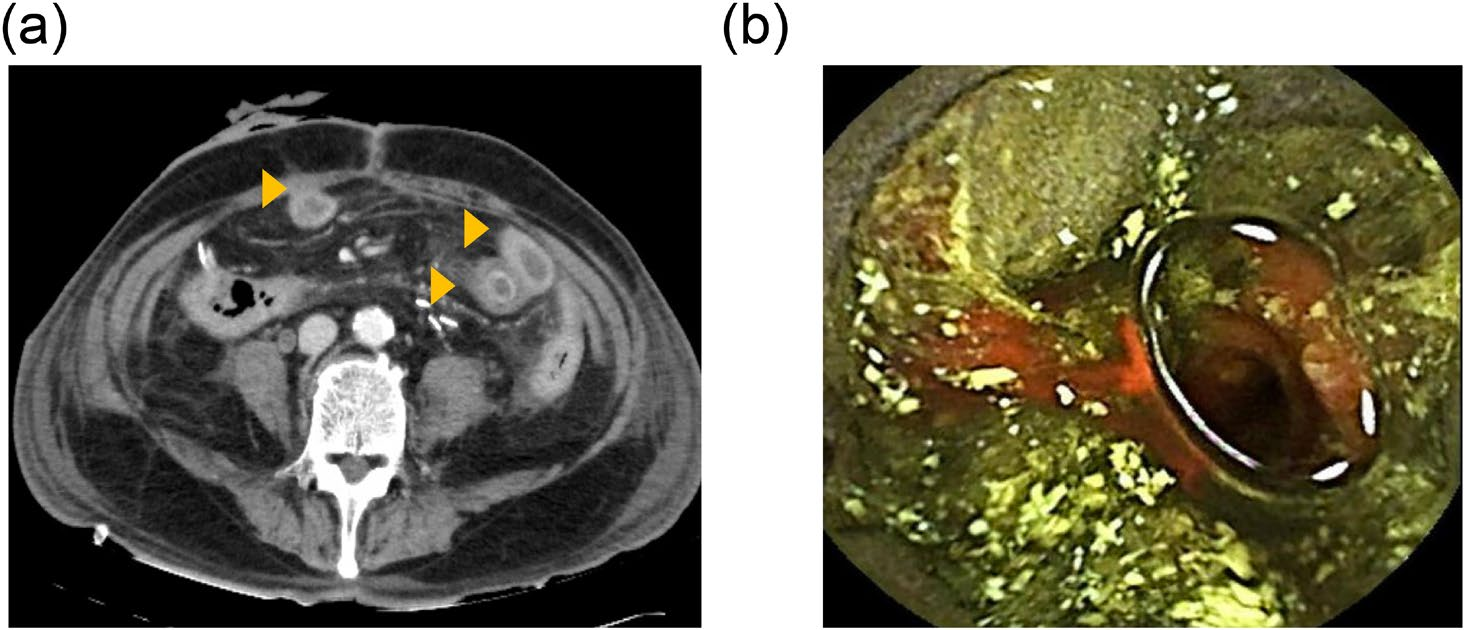
Imaging findings of the small intestine. (a) Contrast‐enhanced abdominal computed tomography showing wall thickening of the small intestine (orange arrows). (b) Capsule endoscopy showing black discoloration of the small intestinal mucosa, suggestive of necrosis.

After the operation, the inflammatory response was mild; however, serum CMV antigen remained detectable. A decrease in platelet count and abnormalities in coagulation function, including elevated D‐dimer levels, were also observed, raising concern for disseminated intravascular coagulation (DIC). On the thirtieth day of hospitalization, abdominal CT revealed wall thickening of the stomach and small intestine, and capsule endoscopy demonstrated black discoloration of the small intestinal mucosa, suggestive of necrosis (Figure 3a,b). On the 35th day of hospitalization, the patient developed sudden, severe abdominal pain caused by small intestinal perforation. Surgical intervention could not be performed because of his poor overall condition, and he died 2 days later. Postmortem analysis revealed Epstein–Barr virus (EBV) infection in biopsy specimens obtained during lower gastrointestinal endoscopy and in the resected colorectal tissue.

## 3 Discussion

Immune‐related enterocolitis occurs in 8%–27% of patients receiving ICIs [2, 3]. It most commonly develops within 4–16 weeks after treatment initiation; however, as in this case, onset more than 1 year after treatment has also been reported (Table 1) [2, 5, 6, 7, 8, 9, 10]. Diarrhea is the most frequent presenting symptom and may be accompanied by bloody stools and high fever. The endoscopic findings of immune‐related enterocolitis vary widely and include characteristic features such as edema, erythema, erosions, and ulcers, sharing some morphological features with those seen in idiopathic inflammatory bowel disease [4]. Erosions have been reported to be most strongly associated with steroid resistance and the need for second‐line treatment with infliximab [11]. Histopathological features of immune‐related enterocolitis include infiltration of CD8‐positive lymphocytes and plasma cells into the mucosal lamina propria, crypt abscess formation, and epithelial apoptosis, all of which were identified in this case [12].

**TABLE 1:** Case reports of immune checkpoint inhibitor‐associated enterocolitis.

	Age	Disease	Immune checkpoint inhibitors	Days until onset of irAE enterocolitis	CTCAE grade	Imaging findings	Endoscopic findings	CMV/EBV infection	Days from onset of irAE to steroid treatment	Steroid dosage per day	Outcomes of irAE enterocolitis
Fujiwara et al. [5]	76	Melanoma	Ipilimumab+Nivolumab	31 days	3	Toxic megacolon	Diffuse erosion and the loss of vascular pattern	−/−	Soon	Prednisone 1 mg/kg	Death
Yoshino et al. [6]	80	Melanoma	Nivolumab	92 days	NE	Intestinal edema	Ulcerative lesions	−/−	28 days	Corticosteroids 0.5 mg/kg	Death
Yoshino et al. [6]	58	Melanoma	Nivolumab	87 days	NE	Intestinal thickening	Edematous and inflamed mucosa	−/−	Soon	Corticosteroids 1 mg/kg	Improvement
Li et al. [7]	68	Endometrial adenocarcinoma	Sintilimab	40 days	3	NE	Extensive mucosal edema	−/−	24 days	Methylprednisolone 1 mg/kg	Improvement
Lu et al. [8]	71	Lung cancer	Durvalumab	27 days	3	NE	Diffuse ulcers with purulent discharge	−/−	23 days	Methylprednisolone 2 mg/kg	Improvement
Sweep et al. [9]	67	Melanoma	Ipilimumab+ Nivolumab	16 days	NE	NE	Mild unspecific colitis	−/−	13 days	Prednisone 1 mg/kg	Improvement
This case	80	Renal cell carcinoma	Nivolumab	55 months	3	Diffuse bowel wall thickening	Ulcerative lesions	+/+	6 months	Prednisone 2 mg/kg	Death

Abbreviations: CMV, cytomegalovirus; CTCAE, common terminology criteria for adverse events classification; EBV, Epstein–Barr virus; irAEs, immune‐related adverse events; NE, not evaluated.

Diseases that should be considered in the differential diagnosis of immune‐related enterocolitis include infectious enterocolitis. CMV‐related enterocolitis typically develops in immunosuppressed states; however, in this case, CMV was detected in biopsy tissue prior to steroid administration. Inflammation is a key driver of CMV reactivation, which occurs through inflammation‐associated signaling, the presence of CMV‐infected immune cells, and their differentiation into permissive cells that support viral replication [13]. Tissue inflammation from immune‐related enterocolitis may therefore initiate CMV reactivation, which can subsequently be exacerbated by immunosuppressive therapy [13]. EBV infection has also been reported in patients with inflammatory bowel disease and is associated with severe symptoms and treatment resistance [14, 15]. In this case, CMV and EBV were thought to coexist in severe immune‐related enterocolitis, with steroid therapy further enhancing these infections. This cascade contributed to ischemic small bowel necrosis and perforation through thrombus formation in the setting of DIC. To the best of our knowledge, this is the first report of irAE enterocolitis combined with CMV and EBV infection (Table 1) [5, 6, 7, 8, 9]. Gastrointestinal mucosal injury resulting from vasculitis and circulatory complications of CMV enterocolitis has been linked to a high mortality rate [13, 14]. In chronic active EBV infection, approximately 25% of cases also develop vascular lesions, which increase tissue factor expression in vascular endothelial cells, promote coagulation activity, and contribute to organ damage and thrombotic microangiopathy [16]. Therefore, in the management of immune‐related enterocolitis, clinicians should routinely monitor patients for concurrent viral infections and initiate prompt therapeutic interventions when such infections are identified. If patients with immunosuppression due to steroid therapy develop CMV or EBV infections, the dosage of immunosuppressive medications should be adjusted. Antiviral agents, such as ganciclovir, are also administered for CMV infection.

Current guidelines recommend the administration of 1–2 mg/kg/day of prednisolone for irAEs, and in many cases, treatment is initiated with 1 mg/kg of prednisolone. However, in this case, since the patient developed severe immune‐related enterocolitis with ulcerative lesions, treatment was initiated with the maximum dose of prednisolone at 2 mg/kg. Although high‐dose steroid therapy improved diarrheal symptoms, the possibility that the immunosuppressive state worsened the viral infection cannot be ruled out. Therefore, it is necessary to consider the dosage and duration of steroid treatment while balancing therapeutic benefits and risks. The patient had also been managed conservatively for approximately 6 months despite persistent diarrhea, and the initiation of steroid treatment was delayed compared with that in other reports (Table 1) [5, 6, 7, 8, 9]. The response rate of steroid treatment for immune‐related enterocolitis varies significantly depending on the Common Terminology Criteria for Adverse Events classification (CTCAE) grade. Cheung and colleagues revealed that while CTCAE diarrhea grade does not correlate with the duration of steroid administration, patients with Grade 3 or higher colitis are significantly more likely to require immunosuppressants such as infliximab due to steroid resistance [11]. Therefore, referral to specialists is recommended if mild symptoms, such as Grade 1 immune‐related diarrhea according to the CTCAE classification, do not improve after more than 1 week [2, 3]. In this case, rectal inflammation was identified on lower gastrointestinal endoscopy soon after the onset of diarrhea. Considering the possibility of immune‐related enterocolitis, a multidisciplinary irAE management team, including a rheumatologist, infectious disease specialist, and other relevant specialists, should have been consulted at this point.

## 4 Conclusions

We encountered a case of immune‐related enterocolitis that progressed to necrosis of the colon and small intestine. Persistent diarrhea during ICI therapy warrants evaluation for immune‐related colitis, even when symptoms appear mild, and should lead to early specialist consultation. Clinicians must also remain vigilant for concurrent infectious enterocolitis, as such complications can exacerbate disease severity and lead to life‐threatening outcomes, including DIC.

## Consent

The authors have nothing to report.

## Conflicts of Interest

The authors declare no conflicts of interest.

## Data Availability

The data that support the findings of this study are available from the corresponding author upon reasonable request.
